# Temperature and Imbibition Influence *Serianthes* Seed Germination Behavior

**DOI:** 10.3390/plants8040107

**Published:** 2019-04-21

**Authors:** Thomas E. Marler

**Affiliations:** College of Natural and Applied Sciences, University of Guam, UOG Station, Mangilao, Guam 96923, USA; thomas.marler@gmail.com; Tel.: +1-671-735-2100

**Keywords:** Physical dormancy, *Serianthes grandiflora*, *Serianthes kanehirae*, *Serianthes nelsonii*

## Abstract

The direct role of physical dormancy in delaying germination of *Serianthes grandiflora* Bentham, *Serianthes kanehirae* Fosberg, and *Serianthes nelsonii* Merrill seeds has not been adequately studied, nor has the role of temperature on germination behaviors. Imbibition testing indicated seeds with scarified testa absorbed water for the duration of a 24 h imbibition period, but seeds with an intact testa stopped absorbing water after 1 h. The behavior of *S. nelsonii* seeds most closely matched those of *S. kanehirae*, with the pattern of water absorption for *S. grandiflora* seeds deviating from that for the other species. Scarified seeds germinated readily, with initial germination occurring by 50 h for *S. nelsonii* and 90 hr for the other species, and maximum germination of 80% to 90% occurring by 60 h for *S. nelsonii* and 100 h for the other species. Predicted optimum temperature based on a fitted quadratic model was 26 °C for *S. nelsonii*, 23 °C for *S. grandiflora*, and 22 °C for *S. kanehirae*. Seed respiration increased within 3 h of imbibition for scarified seeds and continued to increase in a linear pattern. The linear slope was greatest for *S. nelsonii*, intermediate for *S. grandiflora*, and least for *S. kanehirae*, but ultimate respiration was greatest for *S. kanehirae* seeds. Seed respiration was so limited for un-scarified seeds that the instrument was unable to quantify any carbon dioxide efflux. Physical dormancy in seeds of these *Serianthes* species is a powerful trait that spreads out the timing of seedling emergence in natural settings and controls imbibition and germination speed in managed nurseries.

## 1. Introduction

*Serianthes nelsonii* is a critically endangered legume tree species restricted to the islands of Guam and Rota [1]. The populations are not regenerating, and seedlings that emerge in situ exhibit rapid mortality [2,3,4]. Moreover, thousands of propagated individuals will be required to fulfill the goals of the published species recovery plan [5,6], illuminating the mandate to successfully germinate thousands of seeds in managed nurseries. The seed-to-seedling transition is of critical importance, is the initial phenological transition of a sexually-propagated plant, and the underlying requirements for this transition are some of the most important seed traits related to plant fitness [7]. Yet germination behavior of this species has been minimally studied. A greater understanding of *S. nelsonii* germination requirements may aid in understanding the limitations to recruitment in natural settings and improve methods in conservation nurseries.

Research efforts involving endangered plant species within the United States are often limited by handling permit restrictions. For these species, using congeneric species in paired research trials or as surrogates may enable the acquisition of more answers to critical research questions. Using congeneric species is a good way to increase knowledge when seeds of a closely related species are in short supply. The genus *Serianthes* consists of 10 species ranging from the Malay Peninsula to New Caledonia [4,8]. *Serianthes grandiflora* and *S. kanehirae* are the congeneric species that are geographically close to the *S. nelsonii* range, and both have been used to augment nursery research protocols for *S. nelsonii* [5,9]. Studies exploring the imbibition traits and the temperature–germination relationships for these species are missing.

A hard seed coat that imposes physical dormancy on seeds is common among legumes [10,11,12]. For these species, any attempt to understand the seed-to-seedling transition within population regeneration strategies must include the environmental and seasonal conditions that reduce or eliminate the physical dormancy. Similarly, horticultural management of a conservation nursery charged with propagating one of these species cannot be successful without basic knowledge of how to mitigate the physical dormancy of seeds.

My first objective was to determine the speed of water absorption by *S. grandiflora*, *S. kanehirae*, and *S. nelsonii* seeds to more fully understand the role of physical dormancy for denying imbibition. I predicted the pattern would be similar for *S. kanehirae* and *S. nelsonii*, as the proportions and shape of the seeds are similar for these two species. My second objective was to determine the influence of incubation temperature on germination behavior of the same species. I predicted the optimal temperature range would be least for *S. nelsonii* due to its highly restricted latitudinal range in comparison to the other species. My third objective was to characterize the initial seed germination dynamics of the same species immediately after imbibition by quantifying carbon dioxide efflux from seeds. The results may enable more informed conservation decisions during recovery efforts.

## 2. Materials and Methods

### 2.1. Water Absorption

The influence of an intact seed coat on water absorption in *Serianthes* seeds was studied in October 2014 for *S. nelsonii* and April 2015 for the other species. Seeds were stored in ambient conditions on Guam between collection and experimental dates. *Serianthes nelsonii* seeds were collected in Guam in February 2013 (Permit TE-84876A-0), *S. grandiflora* seeds were collected in Bohol, Philippines in March 2015, and *S. kaniherae* seeds were collected in Palau in March 2015.

**Figure 1:**
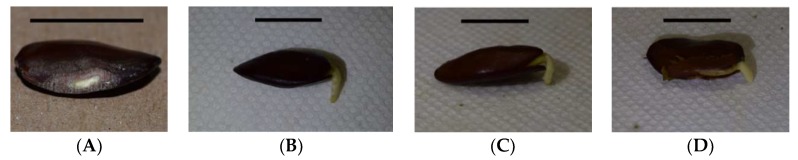
Appearance of *Serianthes nelsonii* seed after scarifying seed coat with sandpaper, exposing the light-colored cotyledon tissue (**A**). The general appearance of (**B**) *Serianthes grandiflora*, (**C**) *Serianthes kanehirae*, and (**D**) *Serianthes nelsonii* seeds when respiration measurements were terminated. Bars = 1 cm.

Water imbibition was determined in scarified versus un-scarified seeds using six replications of five seeds each for *S. grandiflora*, *S. kanehirae*, and *S. nelsonii* seeds. Methods to mitigate physical dormancy followed protocols as previously described [13,14]. Scarification of the seed coat was accomplished by opening a small hole at the midpoint of the long axis of each seed by scratching on 50-grit sandpaper until cotyledon tissue could be seen beneath the testa (Figure 1a). This approach ensured avoidance of the seed location where radicle emergence occurred. Scarified and un-scarified seeds were soaked in aerated water at a constant 24 °C.

At time 0, 0.5 h, 1 h, 1.5 h, then every hour until 24 h, seeds were removed from the water, blotted dry on the surface, weighed, then returned to the water soak treatments. The amount of absorbed water at each time was calculated using the equation: %Wi = ((Wi – Wf) / Wf) × 100, where Wi is fresh weight after water absorption and Wf is initial fresh weight prior to imbibition. The seeds were then germinated for use in a conservation nursery.

The results were plotted to determine the pattern in relation to time of imbibition. The response plots were characterized by linear or cubic regression patterns. The PROC GLM statement in SAS 9.3 (SAS Institute, Cary, Indiana) was used to fit linear and cubic models to each data set. The model with the best fit was selected to define each data set.

### 2.2. Temperature and Germination

The influence of constant temperature on germination of these species was studied in October–December 2014 for *S. nelsonii* and June–August 2015 for the other species. Scarification of seeds was accomplished as described above. Seeds were subsequently soaked in a 10% bleach solution for 3 min, then soaked in tap water for a total of 1 h. Preliminary trials determined that the initial soak with bleach solution did not influence germination of seeds when compared with water only soak. The water was decanted and replaced every 15 min to ensure oxygenation was maintained. Each experimental unit consisted of five imbibed seeds which were sandwiched within wet paper towels and placed in 18 × 19 cm plastic ziploc bags. Germination was checked every 12 h and the paper towels were re-moistened if needed. A seed was recorded as germinated when the radicle reached 1 mm in length, and was removed from the germinating bags and added to a conservation nursery. Germination for each replication continued until every seed germinated or when un-germinated seeds were unmistakably dead. Seeds of these species that do not germinate have an unambiguous appearance and odor.

Seeds were placed in darkness within germination chambers at one of six temperatures within the range of 14 °C to 39 °C. Initial germination time (T_i_) was the time required for the first seed within each replication to germinate. Final germination time (T_100_) was the elapsed time when the last seed to germinate within each replication occurred. Six replications were used for each species and temperature regime.

The germination percentage, T_i_, and T_100_ data were plotted to determine the pattern in relation to incubation temperature. All of the plots were characterized by linear or quadratic patterns. The PROC GLM statement in SAS 9.3 (SAS Institute, Cary, Indiana) was used to fit linear and quadratic models to each data set. The model with the best fit was selected to define each data set.

### 2.3. Imbibition and Seed Respiration

The influence of time since imbibition on germination behavior was studied by quantifying carbon dioxide evolution from seeds. The seed collection dates, experimental dates, and seed preparation protocols were as previously described for each species. An incubation temperature of 24 °C was used, which was close to the optimum temperature for all three species as calculated from the temperature study described above. Following the 1 h imbibition period, carbon dioxide exchange was measured approximately every 3–4 h for the first 48 h, then every 12 h thereafter until the termination of the experiment. The imbibition period was included in the initial 3 h period, such that the initial gas exchange measurements were 2 h after the seeds were removed from the water.

A CIRAS EGM-4 analyzer fitted with an SRC-1 close system chamber (PP Systems, Amesbury, MA. U.S.A.) was used to determine gas exchange. For each replication, the seeds were removed from the paper towel, carefully blotted dry, then remaining surface water was allowed to air dry to reduce condensation risk within the chamber. The five seeds were placed within a ring of modeling clay approximately 10 cm in diameter on a bench surface. The SRC-1 chamber was inserted into the modeling clay to form a sealed chamber of 1.171 L volume. The use of modeling clay to form sealed gas exchange chambers has been previously reported [15]. The EGM-4 recorded air temperature and carbon dioxide change over a 2 min period. This design was tested for leaks by setting up the clay ring on a portable board and inserting the chamber under ambient conditions of 405 ppm carbon dioxide, then moving the board and chamber into a closed laboratory with elevated conditions of 950 ppm carbon dioxide prior to initiating the 2 min recording period. The carbon dioxide within the chamber did not deviate from the initial 405 ppm for the duration of the 2 min period.

Measurements on each replication were stopped when the radicle on any single seed reached 2 mm in length (Figure 1). The germinated seeds were then removed and added to a conservation nursery, and the remaining seeds were allowed to continue germination without further gas exchange measurements. The change in carbon dioxide was divided by five to place the efflux on a per seed basis. The response flux was calculated as mg carbon dioxide per seed per L per min. The seed respiration data were plotted to reveal a linear pattern for every replication as time elapsed following imbibition. The PROC GLM statement in SAS 9.3 was employed to fit the data with linear regression models.

A second respiration study was conducted with *S. grandiflora* and *S. kanehirae* beginning 5 December 2018. The seeds had been in storage at ambient conditions since March 2015. This study was designed to more fully understand the role of physical dormancy on seed behavior. The seed preparation and construction of replications was as described above. There were six replications per species for each of four time periods that were defined by incubation of the seeds without scarifying the testa prior to the 1 h soak in water. The time periods were 0, 2, 4, and 6 weeks of incubation in moist paper towels prior to scarification. Every seed in the experiment was placed in the initial 1 h soak in water, but only the 0 weeks treatment seeds were scarified prior to the water soak. The remainder of seeds were soaked with the testa intact. For the 2, 4, or 6 weeks treatments, the six replications assigned to each time period were removed from the paper towels at the appropriate time, scarified with 50-grit sandpaper, then returned to the moist paper towels without an additional 1 h water soak. Respiration measurements were initiated for each treatment period with time 0 assigned to when the testa was scarified. Every replication in the un-scarified treatments was included in all of the gas exchange measurements in order to quantify seed respiration during adequate germination conditions but without testa scarification.

The responses for each species in each scarification time period were fitted with linear regression models as described above. The influence of duration of seed incubation prior to scarifying the seed testa was determined using repeated measures analysis with the PROC MIXED statement in SAS 9.3. Four response variables were subjected to the analysis: Ultimate germination percentage, the slope of the linear model, measurement temperature, and initial carbon dioxide concentration.

## 3. Results

### 3.1. Water Absorption

**Figure 2:**
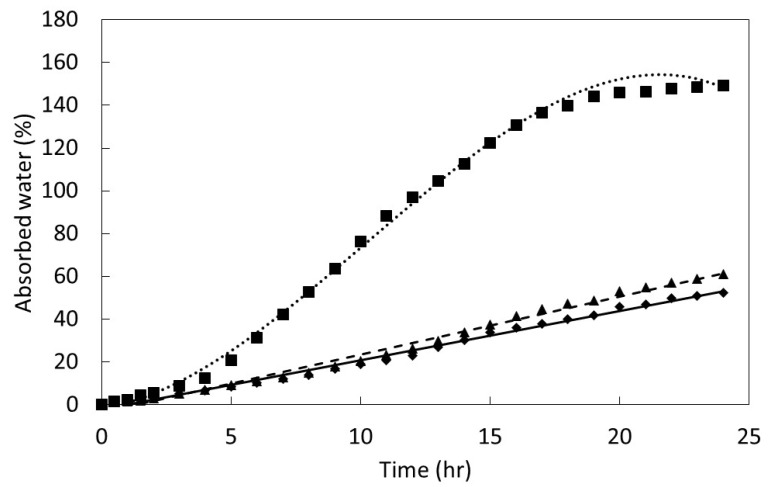
The influence of time in aerated water on water absorption of scarified seeds of three *Serianthes* species, *n* = 6. *Serianthes grandiflora* (squares, dotted line), *y* = −1.57 + 1.93*x* + 0.83*x*^2^ − 0.03*x*^3^, *r*^2^ = 0.98; *Serianthes kanehirae* (triangles, dashed line), *y* = −3.39 + 2.71*x*, *r*^2^ = 0.98; *Serianthes nelsonii* (diamonds, solid line), *y* = −2.27 + 2.31*x*, *r*^2^ = 0.98.

The un-scarified seeds of all three *Serianthes* species absorbed 0.5% water during the first hour of imbibition, then ceased absorbing any more water for the duration of the 24 h period (data not shown). Water absorption of scarified *S. kanehirae* and *S. nelsonii* seeds increased linearly to a maximum of 55% to 60% by the end of the 24 h of imbibition (Figure 2). In contrast, the pattern of water absorption for *S. grandiflora* followed a cubic model with an ultimate absorption of about 150% water.

### 3.2. Temperature and Germination

**Figure 3:**
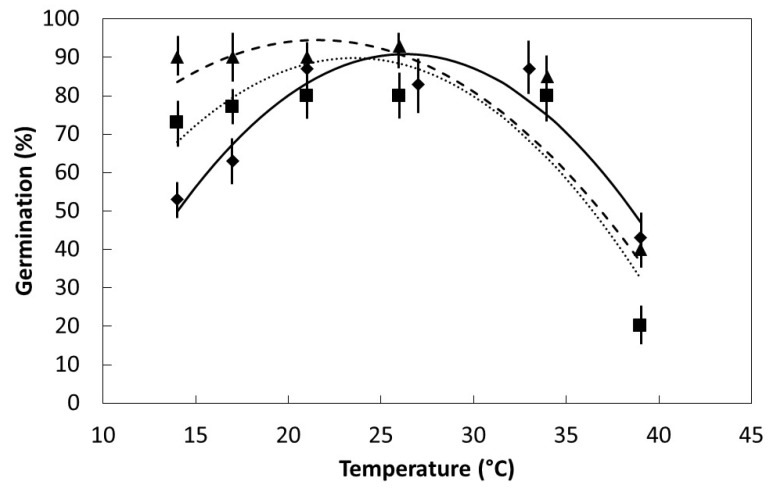
The influence of temperature on germination percentage for *Serianthes* species. Mean ± standard error, *n* = 6. *Serianthes grandiflora* (squares, dotted line), *y* = −43.2 + 11.3*x* − 0.24*x*^2^, *r*^2^ = 0.86; *Serianthes kanehirae* (triangles, dashed line), *y* = 6.0 + 8.2*x* − 0.19*x*^2^, *r*^2^ = 0.90; *Serianthes nelsonii* (diamonds, solid line), *y* = −95.9 + 14.2*x* − 0.27*x*^2^, *r*^2^ = 0.90.

The influence of temperature within the range 14 °C to 39 °C on germination percentage was defined by a quadratic model for all three species (Figure 3). The optimum temperature as defined by the fitted curve was similar among the species, with *S. nelsonii* optimum at 26 °C, *S. grandiflora* optimum at 23 °C, and *S. kanehirae* optimum at 22 °C. The spread in temperature exhibiting similar maximum germination was substantial for the raw data and indicated temperatures between 21 °C and 34 °C would be adequate for germinating all three species. The decline in germination percentage as temperature declined below optimum was greatest for *S. nelsonii*, intermediate for *S. grandiflora*, and was minimal for *S. kanehirae*. In contrast, the decline in germination percentage as temperature increased above optimum was greatest for *S. grandiflora* and similar for the other two species. The span of optimum temperature was least for *S. nelsonii*, as indicated by the greatest linear coefficient and greatest quadratic coefficient for the fitted models.

**Figure 4:**
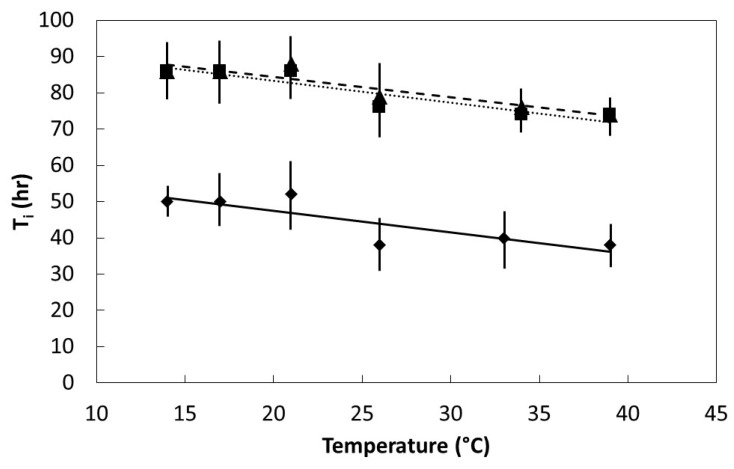
The influence of temperature on initial time of germination for *Serianthes* species. Mean ± standard error, *n* = 6. *Serianthes grandiflora* (squares, dotted line), *y* = 95.6 − 0.6*x*, *r*^2^ = 0.92; *Serianthes kanehirae* (triangles, dashed line), *y* = 95.5 − 0.6*x*, *r*^2^ = 0.74; *Serianthes nelsonii* (diamonds, solid line), *y* = 59.5 − 0.6*x*, *r*^2^ = 0.74.

All three species exhibited a linear decline in T_i_ as temperature increased within the range 14 °C to 39 °C (Figure 4). The negative slope was similar for all three species, but the intercept for *S. nelsonii* was only 62% of that for the other two species, indicating T_i_ was least for *S. nelsonii*. 

**Figure 5:**
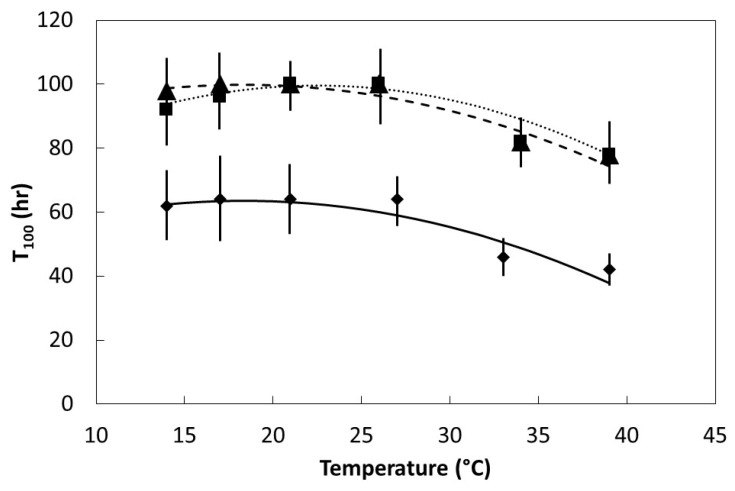
The influence of temperature on final time of germination for *Serianthes* species. Mean ± standard error, *n* = 6. *Serianthes grandiflora* (squares, dotted line), *y* = 59.2 + 3.6*x* − 0.08*x*^2^, *r*^2^ = 0.85; *Serianthes kanehirae* (triangles, dashed line), *y* = 79.8 + 2.2*x* − 0.06*x*^2^, *r*^2^ = 0.88; *Serianthes nelsonii* (diamonds, solid line), *y* = 43.8 + 2.2*x* − 0.06*x*^2^, *r*^2^ = 0.88.

All three species exhibited a quadratic relationship between T_100_ and temperature within the range 14 °C to 39 °C (Figure 5). The linear and quadratic coefficients of the quadratic equations were similar among the species so the shapes of the quadratic models were similar. However, as with T_i_, this response variable was much less for *S. nelsonii* than for the other species.

### 3.3. Imbibition and Seed Respiration

**Figure 6:**
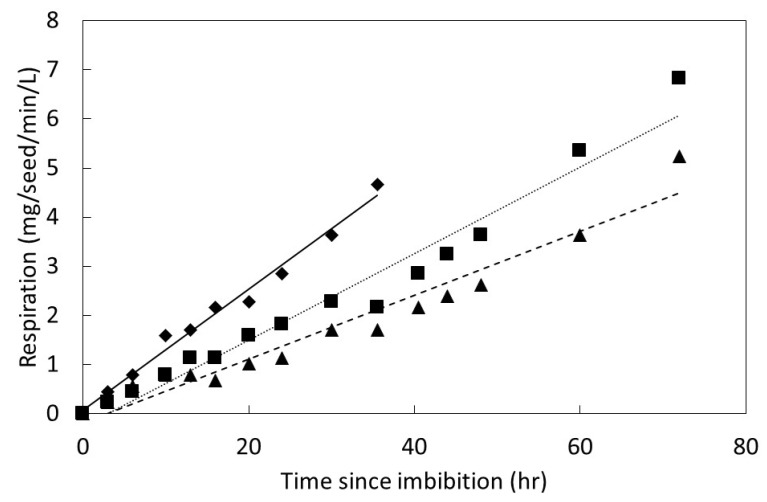
The influence of time since imbibition on seed respiration for *Serianthes* species, *n* = 6. *Serianthes grandiflora* (squares, dotted line), *y* = −0.261 + 0.088*x*, *r*^2^ = 0.96; *Serianthes kanehirae* (triangles, dashed line), *y* = −0.188 + 0.065*x*, *r*^2^ = 0.92; *Serianthes nelsonii* (diamonds, solid line), *y* = 0.082 + 0.123*x*, *r*^2^ = 0.98.

An increase in seed respiration was measured within 3 h of imbibition for all three *Serianthes* species (Figure 6). The 3 h value of 0.45 mg/seed/min/L for *S. nelsonii* was more than double that of the other two species, indicating greater speed in initiating germination. Respiration increased linearly until the measurements were terminated when three seeds within each replication had germinated. This occurred after only 36 hr for *S. nelsonii*, but the other two species required 72 h to reach this stage. The slope of the linear model was greatest for *S. nelsonii*, intermediate for *S. grandiflora*, and least for *S. kanehirae*. The peak respiration was similar for *S. nelsonii* and *S. grandiflora*, and these species exhibited peak respiration which was about 74% of that for *S. kanehirae*.

Seeds that were scarified at 0 weeks in the repeated measures study exhibited seed respiration that was similar to that in Figure 6. However, the seeds that were soaked without scarification did not emit any measured carbon dioxide during the 2 min gas exchange recording period. Seeds incubated in germination conditions prior to scarifying the seed coat for 2, 4, or 6 weeks exhibited increased respiration that was similar in speed and quantity to the 0 weeks treatment immediately after each treatment received the prescribed scarification. The 4 and 6 weeks replications again did not emit any measured carbon dioxide when the 2 weeks scarification treatment measurements were conducted. Similarly, the 6 weeks seeds did not emit any measured carbon dioxide when the 4 weeks scarification treatment measurements were conducted.

The germination percentage for the seeds among the four incubation treatments were not different for *S. grandiflora* or *S. kanehirae* (Table 1). Similarly, the positive linear slope that characterized the increase in seed respiration following scarification did not differ among the four incubation treatments for either species. The mean germination and mean respiration slope were similar to those in Figure 2 (at the same incubation temperature) and Figure 6. These results indicated that incubating in adequate conditions for germination for up to 6 weeks without scarifying the testa did not reduce germination performance immediately after imposing the scarification treatment. Experimental temperature and carbon dioxide concentration did not differ among the four incubation treatments.

## 4. Discussion

The requirements for successful seed germination and early seedling growth are rarely known for native tree species that are not important in silviculture [16]. This limitation in knowledge is amplified for species that are threatened before any empirical research has been conducted. I have begun to address these issues for Guam’s *S. nelsonii*, a native tree that has been reduced to a single mature individual. Seed viability does not appear to be a limitation that explains the acute regeneration failures of *S. nelsonii*. An intact testa allows seeds of this species and the congeneric *S. grandiflora* and *S. kanehirae* to be stored long term by way of physical dormancy, even in germination-appropriate conditions. Ample seedling emergence occurs in situ [3], and extremely rapid metabolic activity and germination of scarified seeds following imbibition has been demonstrated herein. Following a break in the testa, an increase in metabolic activity of the seeds as evinced by seed respiration can be measured in only 3 h of water absorption. These respiration results evince the rapid initiation of *S. nelsonii* germination processes, such as mobilization of storage components and activation of DNA, cell membranes, and organelles [17]. The fairly flat *S. nelsonii* germination response within the temperature range 21 °C to 33 °C (Figure 3) indicates the use of ambient laboratory or nursery settings or air-conditioned laboratory settings would be appropriate for germinating *S. nelsonii* seeds. The increased container substrate temperatures that result from high light conditions [13] mandate the use of darkness or deep shade for germination.

My prediction that the range in optimum temperature for germination would be least for *S. nelsonii* due to its restricted endemic range was confirmed. The span between the two island groups that comprise the *S. nelsonii* endemic range is 60 km. The span between the two island groups that comprise the *S. kanehirae* endemic range is 420 km. The indigenous range of *Serianthes grandiflora* is extensive, encompassing much of Malesia and beyond [18]. The decline in germination within the supra-optimal temperature range was similar among the species, but the decline in germination within the suboptimal temperature range was greatest for *S. nelsonii*. The inhibition of seedling emergence in full sun conditions [13], is now known to be partly due to increased temperatures of the germination medium (up to 44 °C), as temperatures of less than 40 °C reduced germination in the present study. My prediction that water absorption patterns would be similar for *S. kanehirae* and *S. nelsonii* was also confirmed. *Serianthes nelsonii* seeds are much smaller than *S. kanehirae* seeds, but the length, width, and depth of these seeds exhibit similar proportions (*S. kanehirae*: 18.3 × 9.6 × 3.0 mm; *S. nelsonii*: 13.0 × 7.5 × 2.1 mm). In contrast, *S. grandiflora* seeds exhibit much greater depth in relation to length and width (17.8 × 6.8 × 4.3 mm), with cotyledon tissue volume being much greater relative to the seed dimensions. The depth of *S. kanehirae* and *S. nelsonii* seeds is 16% of seed length, but the depth of *S. grandiflora* seeds is 24% of seed length. This contrasting seed shape may explain the highly disparate water absorption curve for *S. grandiflora* in comparison to the other two species. Another notable outcome of this study was the speed of germination for *S. nelsonii* was strikingly faster than for the other species.

Physical dormancy of seeds is common in the Fabaceae family, and some of the natural factors that break down the testa to enable germination are high temperature, fluctuating temperature, and passage through the digestive tracts of animals [11,12]. Thick-walled palisade cells in the testa appear to be a common means by which imbibition of water is denied. The genus *Serianthes* is a member of the Mimosoideae subfamily of Fabaceae. In this subfamily, breaking physical dormancy may also involve a specialized lens [12] or a pleurogram [19] in the testa, both of which may aid initial water absorption. Although the seed coat traits such as lens and pleurogram of other *Serianthes* species have been observed [20], the presence or absence of such features have not been determined for the *Serianthes* taxa in the present study. Continued non-destructive studies are needed to determine the characteristics of all specialized structures in the *S. nelsonii* seed coat and if a minimal scarification of the outer palisade layer rather than scarification of the entire seed coat is sufficient to enable imbibition. Moreover, the use of wet paper towels or filter paper for the imbibition phase rather than full immersion may prove to be adequate.

Seed germination is a complex process that begins as a physical phase during which water is absorbed, then transitions into a metabolic phase where myriad factors combine to eventually lead to radicle protrusion [21,22]. The metabolic phase is still not fully understood, and includes feedback loops and regulatory features such as transcription and epigenetic components that are under hormonal control [23,24,25,26,27]. The speed of imbibition during the physical phase, as shown in Figure 2, may not necessarily correlate directly with the speed of ultimate germination as in Figure 4 and Figure 5, or a direct measure of metabolism as in Figure 6.

Conservation efforts with threatened terrestrial plant species often include germination of seeds to produce plants for recovery purposes and to test viability during long-term seed storage. In order to increase the understanding of germination biology of a threatened model species, these propagation steps can be designed such that non-destructive experiments enable the generation of new information in the process [28]. In this case study, all of the seedlings resulting from germinated *S. nelsonii* seeds were not influenced by the treatments and were added to a conservation nursery to advance recovery efforts.

These results combine with earlier publications [2,3,4,13,14] and observations to contribute toward an understanding of the reasons for natural regeneration limitations. Physical dormancy in seeds of these *S. nelsonii* spreads out the timing of seedling emergence in natural settings and controls germination speed in managed nurseries. Light is not required for germination, and full sun conditions reduce germination. Numerous seedlings emerge beneath mature *S. nelsonii* trees almost every month of the year. However, these seedlings exhibit rapid mortality. In the present study, *S. nelsonii* was shown to exhibit much more rapid germination behaviors than the other *Serianthes* species. This was evident not only in the germination speed, but also in the greater initial seed respiration for *S. nelsonii*. Continued research efforts should focus on the limitations to successful seedling-to-sapling transition (e.g., [29]), as the seed-to-seedling transition does not appear to be a limitation.

## 5. Conclusions

The Recovery Plan for *Serianthes nelsonii* [6] included the critical need for more research to improve management of the species. The results herein contributed directly to that need, and the direct role of physical dormancy in delaying germination of *S. nelsonii* and two other *Serianthes* species has been demonstrated. Intact seed coats denied water absorption under imbibition conditions that enabled 50% to 150% water absorption for scarified seeds. Seed respiration increased within 3 h of imbibition for scarified seeds, evincing a rapid increase in metabolic activity upon imbibition. Scarified *S. nelsonii* seeds initiated germination by 50 h, and the other species did so by 90 h. Optimum germination temperatures and declines in germination within supra-optimum temperatures were similar among the species, but declines in germination within suboptimum temperatures were greatest for *S. nelsonii*. Physical dormancy in seeds of these *Serianthes* species is a powerful trait that spreads out the timing of seedling emergence in natural settings and controls imbibition and germination speed in managed nurseries.

## Figures and Tables

**Table 1:** Parameters from repeated measures analysis comparing the manner in which *Serianthes* germination percentage and slope of the linear increase in seed respiration were influenced by pre-scarification incubation times. Seeds were incubated in wet paper towels for 0, 2, 4, or 6 weeks prior to scarifying the seed coat to enable imbibition, *n* = 6.

Response Variable	*F*-value	*p*-value	Mean
*S. grandiflora* germination (%)	0.5879	0.6323	82.7
*S. grandiflora* linear slope	0.2397	0.8673	0.088
*S. grandiflora* temperature (°C)	0.1402	0.9344	28.9
*S. grandiflora* CO_2_ (ppm)	1.0121	0.4148	408
*S. kanehirae* germination (%)	1.2505	0.3266	89.4
*S. kanehirae* linear slope	0.7101	0.5609	0.066
*S. kanehirae* temperature (°C)	0.2308	0.8735	28.6
*S. kanehirae* CO_2_ (ppm)	0.1897	0.9017	405
